# C-Reactive Protein as a Potential Peripheral Biomarker for High-Lethality Suicide Attempts

**DOI:** 10.3390/life12101557

**Published:** 2022-10-07

**Authors:** Andrea Aguglia, Antimo Natale, Laura Fusar-Poli, Giovanni Battista Gnecco, Alessio Lechiara, Margherita Marino, Matteo Meinero, Fabrizio Pastorino, Alessandra Costanza, Giorgio Alfredo Spedicato, Andrea Amerio, Gianluca Serafini, Eugenio Aguglia, Mario Amore

**Affiliations:** 1 Department of Neuroscience, Rehabilitation, Ophthalmology, Genetics, Maternal and Child Health, Section of Psychiatry, University of Genoa, 16132 Genoa, Italy; 2 Istituto di Ricovero e Cura a Carattere Scientifico Ospedale Policlinico San Martino, 16132 Genoa, Italy; 3 Department of Clinical and Experimental Medicine, University of Catania, 95123 Catania, Italy; 4 Department of Psychiatry, Faculty of Medicine, University of Geneva (UNIGE), 1211 Geneva, Switzerland; 5 Department of Psychiatry, Faculty of Biomedical Sciences, University of Italian Switzerland (USI), 6912 Lugano, Switzerland; 6 Department of Statistics and Quantitative Methods, University of Milano-Bicocca, 20126 Milan, Italy

**Keywords:** inflammation, suicide, c-reactive protein, lethality, biomarkers

## Abstract

The aim of the present study was to identify a cut-off of c-reactive protein (c-RP) potentially predictive of high-lethality suicide attempts (SA) in an inpatient psychiatric sample. After attempting suicide, subjects were admitted to the emergency ward of the IRCCS Ospedale Policlinico San Martino and later to the section of Psychiatry from 1 August 2013 to 31 July 2018. C-reactive protein was measured. The Area Under the Receiver Operating Characteristic (ROC_AUC) was used to assess the discriminative capacity of c-RP for high- vs. low-lethality SA, and a logistic regression was performed to detect the odds ratio, adjusted for age and sex. High-lethality suicide attempters were 133 (30.8%), while low-lethality suicide attempters were 299 (69.2%). The optimal cut-off threshold (and corresponding sensitivity and specificity values) for c-RP was 4.65 mg/L (68/71%). This cut-off corresponds to an AUC of 73.5%. An odds ratio of 4.70 was generated for current high-lethality SA after a logistic regression, adjusted for age and sex. Research on social and biological factors underlying the lethality of SA is crucial for a better understanding of this complex phenomenon. Identifying potential predictors of SA, especially those at high lethality, is essential to implement personalized preventive strategies.

## 1. Introduction

Suicidal behaviors are defined by the Columbia Classification Algorithm of Suicide Assessment as a wide range of behaviors that go from completed suicide—that is, a fatal suicidal behavior—to suicide attempts or ideation—that are nonfatal suicidal behaviors [1].

More than 800,000 people die by suicide every year, and there are more than 20 suicide attempts for each completed suicide. Suicide attempts can occur throughout the lifespan and were the fourth leading cause of death among 15–29-year-olds globally in 2019. The World Health Organization (WHO) recognizes suicide as a public health priority, as it is a global phenomenon in all regions of the world. Suicidal behaviors have a ripple effect that impacts families, caregivers, friends, colleagues, communities, and society, showing long-lasting effects on the people left behind. Therefore, the WHO Mental Health Gap Action Programme was launched in 2008 in order to provide evidence-based technical guidance to scale up service provision and care for mental, neurological, and substance use disorders across countries [2]. It is well known that suicide is a multifaced and multidimensional phenomenon, resulting from the interaction of several biological, genetic, psychosocial, cultural, and environmental factors, as reported by the literature [3,4,5,6,7,8,9,10,11,12]. These risk factors may be divided into distal (i.e., predisposing factors), developmental (i.e., mediating factors), and proximal (i.e., precipitating factors). In particular, distal factors contribute to the risk of suicide but have a distant temporal relationship with the suicide crisis. Among the distal risk factors for suicide, family history, genetic variation, and early life adversity (ELA) are the most studied. Distal factors do not directly trigger suicidal events but predispose them through long-term effects on gene expression and regulation. In turn, mediating factors—which may derive directly from genetic changes due to distal factors or may be associated with other factors such as chronic substance abuse—increase the risk of suicide by accentuating the related behavioral and emotional traits to suicide. These traits can be impulsive-aggressive behavior and anxiety traits. Finally, proximal risk factors, such as depressive psychopathology and acute substance use, often triggered by life events, act as the last factors in a chain of events and can lead to the realization of the suicidal event [3].

The existing literature has extensively demonstrated that the two main risk factors for suicide remain a positive history of a previous suicide attempt and having a family history of suicide [3], even if the assessment of suicide risk based exclusively on the patient’s clinical history has low specificity. In fact, despite the existence of these numerous clinical risk factors, none can give clinicians a certain prediction of the suicide event [13].

In this context, a biological vulnerability could represent one shared element underlying the various risk factors. Therefore, the search for predictive biomarkers is currently considered a real challenge and has grown increasing attention among researchers in the field [13].

Research has identified potential genetic, neuroimaging, and peripheral biomarkers regarding biological factors. As for genetic factors, a relationship between suicide and some specific genetic mutations (i.e., FKBP5, CRH, CRHBP, CRHR1, CRHR2, NR3C1, NR3C2, SKA2, MC2R, and POMC) or epigenetic alterations (i.e., miRNAs) has been reported. Moreover, suicidal behaviors seem related to specific neuroimaging patterns, such as the reduced activity of the prefrontal cortex, microglial density in the anterior cingulate cortex, increased volume and activity of the amygdala, reduced hippocampal volume, and neurotransmitter changes in the raphe nuclei and locus coeruleus. Other studies have reported increased levels of some peripheral biomarkers, such as proinflammatory cytokines and endocannabinoids, or alterations in systems such as the hypothalamic-pituitary-adrenal axis and the serotonergic system [10,14,15,16,17,18,19,20,21,22,23]. Furthermore, central and peripheral immune dysregulation have been proposed as important pathways underpinning the pathophysiology of suicidal behaviors. Specifically, peripheral inflammatory mediators have been identified as promising candidate immune biomarkers. A possible pathway implicating immune-inflammatory processes is through the release of cytokines and their consequences, which have neurotoxic effects, leading to a breakdown of the blood-brain barrier, thereby allowing activated immune cells and their products to influence brain functions. Therefore, research on the role of biological factors in suicidal behaviors could help to clarify the physiopathological basis of this phenomenon and promote the development of early preventive diagnostic models and specific psychopharmacological targets [24].

C-reactive protein (c-RP) is a positive inflammatory protein easily measurable in plasma by highly sensitive assays and synthesized by Kupffer cells in the liver in response to an acute inflammatory event [25,26]. C-RP has been identified as a peripheral biomarker in clinical and translational research; it is indicative of a chronic inflammatory state, depending on value [27]. Over the years, evidence on the role of c-RP as a biomarker to optimize risk stratification and prognosis in major psychiatric disorders has been accumulating [28]. In particular, c-RP could be considered a “psychiatric biomarker” that can alert physicians about chronic inflammatory states, adverse drug effects, patient’s cardiometabolic status, and comorbid conditions. Moreover, c-RP could help clinicians to evaluate better clinical outcomes and choose the optimal treatment selection [28]. A recent systematic review and meta-analysis found a significant association between c-RP levels and suicidality. Furthermore, the authors found that c-RP levels were higher in individuals with high suicidal ideation and in those with suicide attempts compared to nonsuicidal individuals (either patients or healthy controls), without any difference in terms of lethality [29]. However, to our knowledge, no study has attempted to estimate a cut-off to distinguish between high- to low-lethality suicide attempts. The study of suicide attempts with high-lethality is fundamental as they can be considered proxies of completed suicide [30]. Thus, the study and identification of peripheral and central candidate biomarkers may be helpful in disentangling the complexity of suicidal behaviors in order to implement adequate preventive strategies with reduced mortality of patients at high risk and less economic and societal burdens.

Therefore, with the present study, we aimed to evaluate the differences in c-RP levels between high- and low-lethality suicide attempters. Moreover, we attempted to identify a potential cut-off of c-RP to identify those patients who are more at risk of committing high-lethality attempts based on their peripheral inflammatory status.

## 2. Materials and Methods

### 2.1. Study Design and Participants

Our sample of adult patients was recruited at the Section of Psychiatry, Department of Neuroscience, Rehabilitation, Ophthalmology, Genetics, Maternal and Child Health (DINOGMI), Istituto di Ricovero e Cura a Carattere Scientifico (IRCCS) Ospedale Policlinico San Martino, University of Genoa (Italy), from 1 August 2013 to 31 July 2018.

To be included in the study, participants had to fulfill the following inclusion criteria: (a) being hospitalized in an emergency psychiatric unit for a suicide attempt, (b) being 18 years of age or older, and (d) providing written informed consent to participate in the study. The exclusion criteria were as follows: (a) pregnancy or recent childbirth, (b) any condition affecting the ability to fill out the assessment, such as major neurocognitive disorders, (c) any severe neurological disorder or positive history of acute neurological injury, including an intellectual disability, (d) receiving treatment with any class or anti-inflammatory drugs, and (e) the inability or refusal to provide written informed consent to participate in the study.

During the hospitalizations, clinical evaluations were carried out by expert clinicians and carefully reviewed by a senior psychiatrist (with at least 10 years of clinical experience in an inpatient clinical setting and suicidal behaviors). The sociodemographic and clinical characteristics investigated and the method of collection of peripheral venous blood, examined in the laboratory analysis of IRCCS Ospedale Policlinico San Martino, Genoa, Italy, were reported in previously published articles [6,31,32].

The term “suicidal lethality” has not yet been defined outside the health literature. Beyond one publication describing suicide lethality as the lethality of the chosen suicide method [33], some theorists such as Shneidman and Joiner conceptually identified suicide lethality “as a key ingredient of serious suicidality” [34,35]. We adopted Joiner’s definition of suicide lethality, defined as “the acquired ability to enact lethal self-injury” [35]. Within suicide lethality, the only individual intent is to perish as a result of the lethality of self-inflicted actions. Methods of suicide attempts were dichotomized in terms of lethality. Furthermore, a high-lethality suicide attempt was defined as a suicide attempt that warranted hospitalization for at least 24 h and either treatment in a specialized unit (including intensive care unit, hyperbaric unit, or burn unit), surgery under general anesthesia, or extensive medical treatment (beyond gastric lavage, activated charcoal, or routine neurological observations), including antidotes for drug overdoses, telemetry, or repeated tests or investigations. Conversely, a low-lethality suicide attempt was defined as a suicide attempt that did not meet these criteria [6,31,32].

Potential participants were provided with an in-depth explanation of the study objectives and procedures and an opportunity to ask questions. The study was designed in agreement with the guidelines from the Declaration of Helsinki [36] and was approved by the local Ethical Review Board.

### 2.2. Statistical Analysis

Sociodemographic and clinical characteristics of the sample were presented as mean and standard deviation (SD) for continuous variables and as frequency and percentage for categorical variables. The Kolmogorov–Smirnov test was conducted to confirm whether all the investigated sample variables followed the normal distribution.

The Area Under the Receiver Operating Characteristic (ROC_AUC) was used to assess the discriminative capacity of c-RP for high- vs. low-lethality suicide attempts. When modeling binary outcomes, the ROC_AUC is a widely used measure of predictive performance when modeling binary outcome; each point represents one of the possible sensitivity-specificity pairs. Its natural ranges are 0.5 (meaning no discriminative power) to 1.0 (meaning perfect discriminative power). The ROC_AUC curve was used to determine the optimal c-RP cutoffs (and corresponding sensitivity-specificity values) based on the Youden index [37,38]. We interpreted the values of ROC curves according to the classification proposed by Hosmer and Lemeshow [39], as follows: 0.5, no discrimination; 0.7–0.79, acceptable discrimination; 0.8–0.89, excellent discrimination; ≥0.9 outstanding discrimination. The R Statistical software and associated R packages such as pROC and DescTools were used to perform a statistical analysis and statistical significance was assessed using a 5% threshold [40].

Subsequently, a logistic regression was performed to detect the odds ratio related to the significant association between the suboptimal c-RP cut-off and high-lethality SA, adjusted for age and sex. The Statistical Package for Social Sciences (Version 25.0, SPSS; SPSS Inc., Chicago, IL, USA) was used for statistical analyses. A *p*-value < 0.05 (two-tailed) was regarded as statistically significant.

## 3. Results

### 3.1. Characteristics of the Sample

A total sample of 432 participants admitted for a suicide attempt was included in the present study. High-lethality suicide attempters were 133 (30.8%), while low-lethality suicide attempters were 299 (69.2%).

Regarding sociodemographic variables, 333 patients were females (77.1%), with a mean age of 49.13 ± 20.16 years, and most frequently singles (45.1%). Patients were most commonly diagnosed with mood disorders (N = 288, 66.7%). The mean of c-RP levels was 13.52 ± 27.86 mg/dL. As reported in our previous work on the same sample [31], c-RP was significantly higher in psychiatric inpatients with high-lethality suicide attempts compared to low-lethality suicide attempters (24.18 ± 38.69 vs. 8.78 ± 19.66).

### 3.2. C-Reactive Protein: Optimal Cut-Off and Accuracy

**Figure 1:**
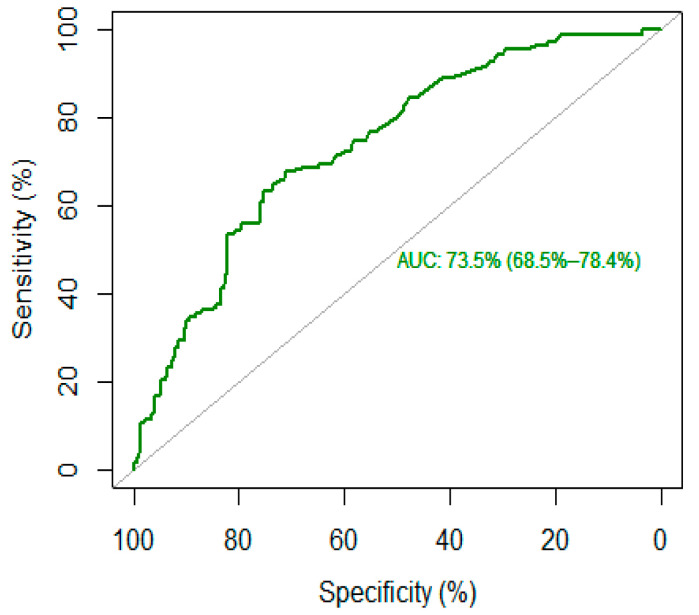
ROC curve of c-reactive protein (c-RP) as a biomarker for high-lethality suicide attempt.

The optimal cut-off threshold for c-RP was 4.65 mg/L, corresponding to a sensitivity of 68% and a specificity of 71%, with higher c-RP levels corresponding to high-lethality suicide attempts (see Figure 1). This cut-off corresponds to an AUC of 73.5% (95% CI: 68.5–78.4%), which reflects an acceptable accuracy of c-RP as a putative biomarker.

After dividing the sample according to the optimal c-RP cut-off value, we conducted a binary logistic regression, adjusted for age and sex, which generated an odds ratio (OR) of 4.70 for current high-lethality suicide attempts (*p* < 0.001; 95% CI: 2.99–7.38). This result indicates that patients with c-RP higher than 4.65 mg/L were more likely to be high-lethality suicide attempters.

## 4. Discussion

Suicide is a complex phenomenon that may be related to several underlying social and biological factors. Over the last years, the role of inflammation as a potential biomarker of suicidal behaviors has gained increasing interest. In the present paper, we evaluated the levels of c-RP in a sample of 432 psychiatric inpatients who attempted suicide and estimated a cut-off to discriminate between high- and low-lethality suicide attempts.

Our data showed that c-RP is increased in high-lethality compared to low-lethality suicide attempts, suggesting higher inflammatory levels in the former group. One possible explanation is that people with high-lethality suicide attempts may present certain behavioral traits, such as impulsivity and aggression [41,42,43]. These traits have been, in turn, associated with increased inflammatory levels. For instance, c-RP levels appeared positively associated with trait aggression and hostility in individuals with personality disorders [44] and with aggressive behavior in individuals diagnosed with intermittent explosive disorders [45]. Moreover, Isung et al. found that plasma levels of interleukin-6 (IL-6)—a cytokine that induces the hepatic synthesis of c-RP—were positively related to impulsivity traits and with violent suicide attempts methods [46]. Furthermore, higher levels of proinflammatory cytokines have been found in the cerebrospinal fluid of patients with a history of violent suicide attempts and those with completed suicide compared to low-lethality attempters [47]. Overall, these findings suggest that systemic inflammation may influence suicidal behaviors in predisposed individuals.

The main element of novelty introduced by our study is the calculation of a putative c-RP cut-off to identify people at higher risk of making a high-lethality suicide attempt. A ROC curves analysis identified a c-RP value of 4.65 mg/mL as the optimal cut-off to discriminate between high- and low-lethality suicide attempts, with an acceptable AUC (73%). A subsequent logistic regression found that people with a c-RP value that exceeded the proposed cut-off were 4.7 times more likely to be classified as high-lethality suicide attempt after adjusting for age and sex. Similarly, in a large prospective cohort study, Batty et al. found that people in the highest inflammation group (c-RP > 3 mg/L) were four times more likely to die by suicide relative to those in the lowest group (c-RP < 1 mg/L). However, in Batty’s paper, the classification was done a priori without any estimation of the optimal threshold [48].

According to our classification, a high-lethality suicide attempt requires hospitalization for at least 24 h with either treatment in a specialized unit, surgery under general anesthesia, or extensive medical treatments [34,35], with consequent high costs for society. Moreover, a first suicide attempt with high-lethality is considered a strong predictor of relapse [32,49]. A high-lethality suicide attempt can be considered a close proxy of completed suicide. Therefore, the study of its neurobiology may shed light on the pathophysiology of suicide [30]. Identifying people who are more at risk of attempting suicide with a high-lethality method is thus crucial, not only for the physical implications but also for a better understanding of suicidal behaviors. In our previous works, we showed other biological differences between high- and low-lethality suicide attempts, such as significantly lower cholesterol levels [31] and higher mean platelet volume and platelet-to-lymphocyte ratio [17]. However, to the best of our knowledge, this is the first study to estimate a potential cut-off for c-RP levels.

Although our findings need to be cautiously interpreted due to the relatively low ROC_AUC value, they might be relevant from a clinical point of view. In fact, c-RP is one of the most widely utilized assays in medicine whose levels can be measured at a relatively low cost through a routine blood examination [29]. Therefore, inserting a periodical c-RP evaluation in routine blood tests could be useful for those who are more at risk of committing suicide (e.g., patients suffering from severe major depressive disorder, people in the depressive phase of bipolar disorder, or presenting with high hostility-impulsivity-aggression traits). It is worth noting that c-RP is a nonspecific marker that can be altered for several reasons, including medical comorbidities that frequently affect people with psychiatric disorders. Therefore, its standalone utility as a biomarker of high-lethality suicide attempts is scarce. However, the evaluation of c-RP levels in association with other clinical red flags may help clinicians prevent patients’ from acting out by implementing adequate strategies.

Of note, c-RP can be considered a valid alternative to cytokines in studies because of its longer half-life and detectability at lower levels [50]. Therefore, its utilization in research settings should be promoted. Future studies should integrate the evaluation of inflammatory biomarkers such as c-RP with a detailed clinical characterization of patients in order to test powerful predictive models of suicide attempts and related lethality. In addition, longitudinal studies may clarify whether there is a cause-effect relationship between c-RP and the lethality of suicide attempts.

Despite the importance of our findings, some limitations need to be acknowledged. First, the cross-sectional design of the present study does not allow for inference of any causal relationship between c-RP levels and the lethality of suicide attempts. Second, we did not take into account some confounding factors potentially affecting c-RP levels, such as body mass index or the psychotropic medications taken by participants. Third, we included only a sample of inpatients; thus, we cannot generalize our findings to psychiatric outpatients. Finally, we did not stratify the analyses according to the type of diagnosis. Nevertheless, it has been argued that c-RP may represent a transdiagnostic biomarker for suicidal behaviors [29]. Finally, no ad-hoc evaluation scale, such as the Columbia Suicide Severity Rating Scale, was used to characterize and better define the suicidal behaviors of participants. In the future, longitudinal studies with periodical follow-ups of c-RP levels combined with the administration of standardized tools may clarify the relationship between peripheral inflammation and suicidal behaviors in people with psychiatric disorders.

In conclusion, our data showed that higher c-RP levels are associated with high-lethality suicide attempts, with people having c-RP levels ≥4.65 mg/L being 4.7 times more at risk. Research on social and biological factors underlying the lethality of suicide attempts is crucial for a better understanding of this complex phenomenon. Therefore, identifying potential biomarkers as predictors of suicide attempts, especially those at high lethality, is essential to implement personalized preventive strategies and identify new pharmacological treatment targets with a central role in the pathophysiology of suicidal behaviors.

## Data Availability

The data presented in this study are available on request from the corresponding author. The data are not publicly available due to privacy/ethical restrictions.
